# Effects of reward contingencies on brain activation during feedback processing

**DOI:** 10.3389/fnhum.2014.00656

**Published:** 2014-08-26

**Authors:** Yi Jiang, Sung-il Kim, Mimi Bong

**Affiliations:** Department of Education, Brain and Motivation Research Institute (bMRI), Korea UniversitySeoul, South Korea

**Keywords:** reward contingency, ventral striatum, amygdala, orbitofrontal cortex (OFC), functional magnetic resonance imaging (fMRI)

## Abstract

This study investigates differential neural activation patterns in response to reward-related feedback depending on various reward contingencies. Three types of reward contingencies were compared: a “gain” contingency (a monetary reward for correct answer/no monetary penalty for incorrect answer); a “lose” contingency (no monetary reward for correct answer/a monetary penalty for incorrect answer); and a “combined” contingency (a monetary reward for correct answer/a monetary penalty for incorrect answer). Sixteen undergraduate students were exposed to the three reward contingencies while performing a series of perceptual judgment tasks. The fMRI results revealed that only the “gain” contingency recruited the ventral striatum, a region associated with positive affect and motivation, during overall feedback processing. Specifically, the ventral striatum was more activated under the “gain” contingency than under the other two contingencies when participants received positive feedback. In contrast, when participants received negative feedback, the ventral striatum was less deactivated under the “gain” and “lose” contingencies than under the “combined” contingency. Meanwhile, the negative feedback elicited significantly stronger activity in the dorsal amygdala, a region tracking the intensity and motivational salience of stimuli, under the “gain” and “lose” contingencies. These findings suggest the important role of contextual factor, such as reward contingency, in feedback processing. Based on the current findings, we recommend implementing the “gain” contingency to maintain individuals’ optimal motivation.

## Introduction

Motivation, a major determinant of behavior, helps individuals to engage in goal-directed behaviors (McClelland, 1985). Furthermore, motivation is an interactive process that can be influenced by external factors such as reward and punishment which are essential in reinforcement learning (Ormond, 1999). The accurate evaluation of performance outcome and the appropriate emotional and motivational reactions toward feedback are crucial for optimal behavior.

A wealth of neuroimaging studies have identified several key brain regions associated with reward, motivation, and emotion, including the ventral striatum, the orbitofrontal cortex (OFC), and the amygdala (see Davis and Whalen, 2001; Kringelbach and Rolls, 2004; Delgado, 2007; Kim, 2013 for review). The ventral striatum has been consistently found to be responsive to both primary (e.g., food) and secondary (e.g., money) reward stimuli (Delgado et al., 2000; Knutson et al., 2001a; O’Doherty et al., 2002). There is also evidence that the ventral striatum is sensitive to the magnitude of a reward (Elliott et al., 2000) and thus considered a primary brain region for the coding of reward and hedonic experience. The OFC is another core brain region responsible for processing reward value (O’Doherty et al., 2001; Rilling et al., 2002; Kringelbach et al., 2003). Researchers have found that the OFC plays a major role in value coding and it responds to the stimuli with the relatively higher value rather than to the absolute value of the stimuli (Tremblay and Schultz, 1999). In addition, empirical evidence also suggest a medial-lateral distinction within the OFC; the medial OFC generally responds to reward whereas the lateral OFC is more sensitive to punishment (see Kringelbach and Rolls, 2004 for a review). Just as important as the ventral striatum and the OFC, the amygdala is known to be involved in both emotion and reward processing (see Davis and Whalen, 2001; Haber and Knutson, 2010 for review). In general, the amygdala interacts with the ventral visual stream and the OFC and evaluates the contextual information to guide decisions (Murray, 2007). In particular, researchers found that the amygdala activity is specifically associated with the framing effect as the amygdala shows increased activity to the safe option in the gain frame and the risky option in the lose frame, indicating its critical role in adjusting individuals’ motivational responses under different conditions (De Martino et al., 2006).

Evidence also revealed that reward-sensitive brain regions are highly context-dependent (Holroyd et al., 2004; Nieuwenhuis et al., 2005). For example, Nieuwenhuis et al. (2005) found differential brain activations in response to the same financial outcome (e.g., $0) under different contexts (i.e., “win” context in which $0 is the worst possible outcome; “lose” context in which $0 is the best possible outcome). The authors concluded that the value of a particular stimulus or event is determined by the context in which it occurs. However, previous studies on reward have typically examined contextual effect by manipulating the relative value of the stimulus. According to Tversky and Kahneman’s (1981) prospect theory, the coding of gains and losses is also greatly influenced by the way in which they are framed. In the economic and business domain, such frame effect is widely applied in terms of the bonus-malus system (BMS). The bonus frame emphasizes the potential reward whereas the malus frame emphasizes the potential penalty. Similarly, Higgins (1997, 1998) proposed two self-regulatory focuses as motivational systems: promotion focus which is sensitive to the signal of gain and prevention focus which is sensitive to the signal of loss. He argued that promotion focus leads to eagerness whereas prevention focus leads to vigilance (Higgins, 2000). Researchers have found that incidental priming of promotion goals activated the OFC whereas the incidental priming of prevention goal activated the anterior cingulate cortex (Eddington et al., 2007).

In our opinion, an identical reward-related feedback may be perceived differently depending on whether the context is promotion-focused (gain context) or prevention-focused (lose context), resulting in differential emotional and motivational responses. For example, in a behavioral study comparing the effects of three reward contingencies on performance satisfaction of middle school students, Bong and Kim (2006) showed that the impact of feedback was dependent on the reward contingency condition. The middle school students reported higher level of performance satisfaction when they experienced a “gain” contingency (a monetary reward for correct answer/no monetary penalty for incorrect answer) than they did in a “lose” contingency (no monetary reward for correct answer/a monetary penalty for incorrect answer) or a “combined” contingency (a monetary reward for correct answer/a monetary penalty for incorrect answer). Yet, little is known about the regional differences in brain activity across various reward contingencies during feedback processing.

In the present study, we aimed to investigate how reward contexts with different focuses (e.g., promotion or prevention) would influence the neural responses during feedback processing. We manipulated three different types of reward contingencies (the “gain”, “lose”, and “combined” contingencies) and compared the brain activities in various regions of interest (ROI) (ventral striatum, OFC, and amygdala) during feedback processing. We expected different brain activation patterns in response to feedback depending upon the type of reward contingency.

## Material and methods

### Participants

The study was approved by Korea University’s Institutional Review Board for human participants. A total of sixteen healthy, right-handed undergraduate students from several private universities in Seoul (seven males, mean age = 22.4, *SD* = 2.28) volunteered to participate in this study via online postings. All participants reported no prior experience with neurological experiments and neurological or psychiatric disorders, and signed a written consent form according to the protocols of Korea University’s Institutional Review Board. All participants received a monetary reward (Korean Won (KRW) 30,000, equivalent to $27) after completing the experiment.

### Experiment design

**Figure 1:**
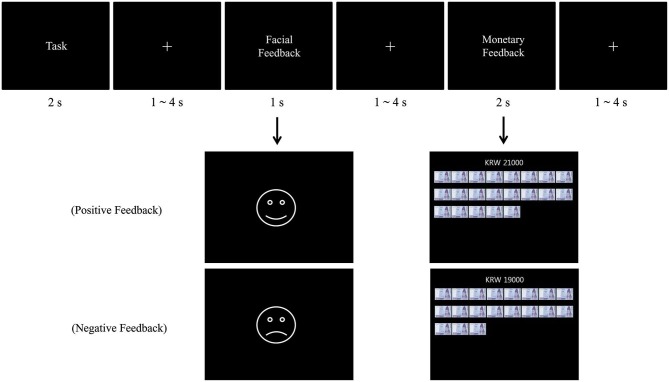
**Trial sequence and feedback patterns**. The first two crosses stand for the jitter between task and feedback, and the last cross represents inter-trial interval.

We used a mixed blocked/event-related design, with three independent runs (one each for the “combined,” “gain,” and “lose” contingencies) during scanning. Each contingency consisted of 40 trials, yielding 120 trials in total. Each trial lasted 11 s, with each contingency lasting 7 min and 20 s. The entire scan took 22 min. For each trial, the task stimulus was presented for 2 s, followed by a random fixation. The durations of the fixation were determined by a sequence of random numbers generated by Excel, ranging from 1 to 4 s with an interval of 0.5 s. The average duration of fixation was 2 s. After the random fixation, the participants received facial feedback for 1 s, notifying them of whether they had succeeded or failed. After another random fixation, participants received monetary feedback that lasted 2 s. This was followed by a random inter-trial interval also ranging from 1 to 4 s (average of 2 s; see Figure 1 for the trial sequence).

The experimental design was programmed and presented using E-PRIME v. 1.1. software. Before scanning, participants performed practice trials to familiarize themselves with the task and the response procedure. During the scanning, participants performed the task in the fMRI scanner through the rear-projection monitor. They were asked to judge whether the number of the stimuli on the screen met the criterion by clicking either the left button (if they believed that the number met the criteria) or the right button (if they believed that the number did not meet the criteria) on a keyboard connected to the fMRI machine. To prevent the practice effect as well as fatigue effect from repetitive performance of the same tasks, we incorporated slightly different tasks into each contingency. Specifically, during the “combined” contingency, participants looked at a computer screen filled with 14 to 16 figures for 2 s. The task was to determine whether the number of figures on the screen was 15 or not. Similarly, participants were asked to detect whether or not the number of letters was 16 during the “gain” contingency and to determine whether or not the number of digits was 16 during the “lose” contingency. The pilot test showed that there was no difference among these tasks in terms of task difficulty and task interest.

Three patterns of feedback were used in accordance with the three types of reward contingencies. Under the “combined” contingency, participants started with KRW 20,000. For a correct answer, an image of a smiley face was accompanied and followed by monetary feedback indicating that the participant received KRW 1000. For an incorrect answer, on the other hand, an image of a sad face was accompanied and followed by monetary feedback indicating that the participant lost KRW 1000. Under the “gain” contingency, participants started with KRW 0. For a correct answer, a smiley face was presented and followed by monetary feedback indicating that the participant received KRW 1000. For an incorrect answer, a sad face was presented and followed by monetary feedback indicating no monetary change. Under the “lose” contingency, participants started with KRW 40,000. For a correct answer, a smiley face was presented and followed by monetary feedback indicating no monetary change. For an incorrect answer, a sad face was presented and followed by monetary feedback indicating that the participant lost KRW 1000. Figure 1 shows a sample of the feedback display.

Unbeknownst to the participants, the feedback was predetermined regardless of their actual performance and all of the participants received identical feedback throughout the whole experiment. We used this method to prevent potential large differences in performance among the participants so that they could end up with the same amount of rewards within each contingency. The success rate was set as 50% for all three contingency conditions, and the sequence of positive and negative feedback was randomized within each contingency. Because the task stimulus was presented for a relatively short period of time (2 s), the high level of task difficulty made the bogus performance feedback credible to the participants. In their post-scanning interviews, all participants reported that they believed the feedback was based on their actual performance. After they had finished the whole experiment, participants were fully debriefed.

For the run sequence, the “combined” contingency was always presented first because it creates a reward context with no specific focus (neither promotion nor prevention). Therefore, it was used as a baseline condition. For the purpose of counterbalancing the order effect of the “gain” and “lose” contingencies, half of the participants were scanned in a “combined-gain-lose” sequence and the other half were scanned in a “combined-lose-gain” sequence.

### Imaging data acquisition

The experiment was conducted at Ewha Womans University Mokdong Hospital. Images were acquired using a Philips Intera Achieva 3T MRI scanner (Philips Medical Systems, Andover, MA, USA). Functional data were obtained using a single-shot gradient echo-planar imaging (EPI) sequence (TR = 2000 ms, TE = 30 ms, flip angle = 90°, field of view (FOV) = 240 mm, ascending, 36 3-mm-thick slices, with no gap). After the first run, high-resolution T1-weighted three-dimensional volumes were acquired for anatomical localization (TR = 9.8 ms, TE = 4.6 ms, 160 slices, voxel size = 1 × 1 × 1 mm).

### Imaging data analysis

Imaging data were preprocessed and analyzed by Statistical Parametric Mapping (SPM 5, Department of Cognitive Neuroscience, London, U.K.) in the Matlab (Mathworks Inc., USA) environment. During preprocessing, functional images were first realigned to the first volume to compensate for subtle head motions. All participants’ head motions were less than 3 mm in any translation within each run. Data were then corrected for differences in timing of slice acquisition, normalized to EPI templates implemented in the SPM, and spatially smoothed using an 8 mm full width at half maximum isotropic (FWHM) Gaussian kernel.

After the preprocessing, statistical analyses were performed on each participant’s data using a general linear model (GLM) in SPM. The analyses were performed by modeling facial feedback (success and failure events for each contingency), monetary feedback (success and failure events for each contingency), and task stimuli as regressors. Participants’ response times during task phases and realignment parameters were also included as regressors in the statistical model. Changes in the Blood-Oxygen-Level-Dependent (BOLD) signal were assessed by linear combinations of the estimated GLM parameters (beta values). Because participants were informed of the contingency condition before they started each run, the present study particularly focused on comparing the facial feedback phase. In other words, once the participants received facial feedback, they were automatically aware that they would receive reward, lose money, or obtain no monetary reward/penalty.

Individual contrast images were estimated by contrasting the beta value of the positive and negative facial feedback against the implicit baseline within each contingency. Thus six types of contrast images were estimated. All individual contrast images were then collected to further examine the statistical significance of the evoked hemodynamic response in a second level random effects analysis. We first conducted a whole-brain 2 × 3 factorial ANOVA with feedback (positive or negative feedback) and contingency (“combined”, “gain”, or “lose” contingency) as factors to test the main effects of the feedback and the contingency as well as the potential interaction of the two factors on brain activation. We also conducted two separate one-way ANOVAs with each feedback valence as factors to explicitly test how positive and negative feedback may recruit distinct patterns of brain activation under various contingencies. The statistical criterion was set at *p* < 0.05 false discovery rate (FDR) corrected for multiple comparisons at the voxel level, with an extent threshold of 10 contiguous voxels. Activations in *a priori* ROI but failed to survive in the whole-brain correction were then subjected to a small-volume correction (SVC). ROI masks for SVC were created based on *a* *priori* anatomical structures rather than observed activation from present result. Specifically, the masks for the bilateral ventral striatum were created as 8-mm spheres centered on the coordinates (Montreal Neurological Institute (MNI) coordinates: *x*, *y*, *z* = −10, 12, −6; 16, 12, −12) indentified in a quantitative meta-analysis which tested the role of the ventral striatum in reward processing (Diekhof et al., 2012). The two coordinates were selected from the activation likelihood estimation (ALE) meta-analysis of reward-related activations when individuals received reward. Similarly, the mask for the left dorsal amygdala was also created as an 8-mm sphere centered on the coordinate (MNI coordinates: *x*, *y*, *z* = −30, −10, −10) reported in a previous empirical study which specifically investigated the role of the dorsal amygdala subregion in modulating attentional resources during emotional processing (Morris et al., 2001).

We then conducted functional ROI analyses and a series of *post hoc* analyses using the least significant difference method with adjusted alpha levels of 0.05 to quantify the mean beta value and activation patterns of the significantly activated brain regions. Functional ROIs were defined by the full cluster of activated regions and were analyzed with the Marsbar toolbox (Brett et al., 2002) in SPM 5. The mean beta values of ROIs were extracted for each participant separately and then averaged for various conditions. The anatomical locations of significant activation foci were determined by using the Talairach and Tournoux (1988) standard stereotaxic space and Duvernoy (1991) atlas.

## Results

### Behavioral results

Participants’ average response times were 1326.88 ms (*SD* = 236.90) in the “combined” contingency, 1399.64 ms (*SD* = 220.05) in the “gain” contingency, and 1392.81 ms (*SD* = 224.44) in the “lose” contingency. A one-way ANOVA revealed no significant difference in response times among the three reward contingencies (*F*_(2,33)_ = 0.089, *p* = 0.92). Likewise, the average missing frequencies (a failure to respond within 2 s of task stimulus time) were 2.67 times (*SD* = 4.38) in the “combined” contingency, 2.67 times (*SD* = 3.75) in the “gain” contingency, and 2.08 times (*SD* = 4.12) in the “lose” contingency. There was also no significant difference among the three reward contingencies (*F*_(2,33)_ = 0.081, *p* = 0.92).

### Imaging results

**Figure 2:**
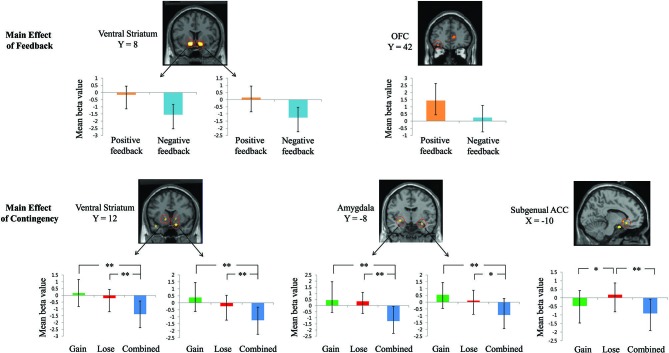
**Results from 2 × 3 factorial ANOVA analysis**. Error bars indicate standard error. **p < 0.01; *p < 0.05.

First, the 2 × 3 factorial ANOVA revealed a significant main effect of feedback on brain activation in the bilateral ventral striatum, the OFC, the anterior cingulate cortex, and the inferior parietal lobule (*p*_FDR-corr_ < 0.05, whole-brain corrected). Functional ROI result indicated that the positive feedback elicited significantly higher activation in the ventral striatum than the negative feedback (see Figure 2). This result indicates that the reward manipulation used in the present study was successful. The positive feedback also elicited significantly greater OFC activation than the negative feedback (see Figure 2).

There was also a significant main effect of reward contingency on brain activation in *a priori* ROIs including the bilateral ventral striatum and the bilateral amygdala (*p*_FDR-corr_ < 0.05, whole-brain corrected). Functional ROI analyses revealed that the ventral striatum showed positive activation only under the “gain” contingency during the overall feedback processing (see Figure 2). Moreover, *post hoc* tests indicated that the ventral striatum was more activated under the “gain” contingency (left: *t*_(15)_ = 5.06, *p* < 0.01; right: *t*_(15)_ = 5.97, *p* < 0.01) and was less deactivated under the “lose” contingency (left: *t*_(15)_ = 4.07, *p* < 0.01; right: *t*_(15)_ = 4.56, *p* < 0.01) than under the “combined” contingency (see Figure 2). The amygdala, on the other hand, was significantly more activated under both the “gain” (left: *t*_(15)_ = 4.30, *p* < 0.01; right: *t*_(15)_ = 4.21, *p* < 0.01) and the “lose” (left: *t*_(15)_ = 4.87, *p* < 0.01; right: *t*_(15)_ = 2.65, *p* = 0.02) contingencies than under the “combined” contingency during the overall feedback processing (see Figure 2). Besides the aforementioned two ROIs, we also found significant activation in the subgenual cingulate gyrus (sgACC, *p*_FDR-corr_ < 0.05, whole-brain corrected), a key brain region involved in perceiving and evaluating emotional stimuli. Specifically, the sgACC showed significantly higher activation under the “lose” contingency than under the “gain” (*t*_(15)_ = 3.32, *p* = 0.01) and the “combined” (*t*_(15)_ = 5.16, *p* < 0.01) contingencies during the overall feedback processing (see Figure 2). There was no significant interaction effect of feedback and contingency on the brain activation in the factorial ANOVA. Table 1 presents detailed information about the activated brain regions from the 2 × 3 factorial ANOVA analysis.

**Table 1:** **Activated brain regions from 2 × 3 factorial ANOVA analysis**.

Brain regions	BA	R/L	Cluster	MNI Coordinates			*z*-value
				*x*	*y*	*z*	
**Main effect of feedback**							
Ventral striatum		L	292	−12	8	−12	5.90
		R	319	12	8	−12	5.59
Anterior cingulate cortex	32	R	81	12	42	6	4.22
	32	R		4	46	2	3.78
	32	R		12	38	16	3.58
OFC	11	L	17	−34	42	−16	4.14
Inferior parietal lobule	40	L	17	−44	−56	42	3.90
**Main effect of contingency**							
Ventral striatum		L	69	−14	14	−2	4.07
		R	12	12	14	−8	3.89
		R	27	16	12	−20	4.60
Amygdala		L	45	−30	−8	−8	4.44
		R	16	30	−8	−18	4.41
Uncus	34	L	126	−16	4	−22	5.22
Putamen		L	69	−20	−2	0	4.39
Superior temporal gyrus	22	L	63	−42	6	−24	4.38
Cerebellum			30	0	−52	−8	4.36
sgACC	25	L	12	−10	24	−12	4.34
Inferior temporal gyrus	19	L	14	−52	−62	0	4.34
Precentral gyrus	4	R	13	40	−26	68	3.99
Inferior frontal gyrus	44	R	14	50	16	12	3.88

*Note. All regions survived at p_FDR-corr_ < 0.05, whole-brain corrected. BA: Brodmann’s area; R/L: right or left hemisphere*.

**Figure 3:**
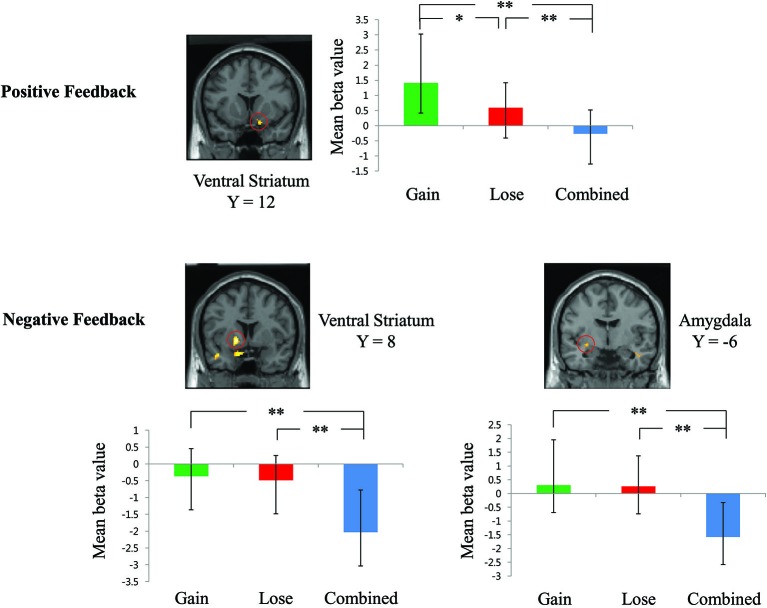
**Results from separate one-way ANOVA analyses with different feedback valence**. Error bars indicate standard error. **p < 0.01; *p < 0.05.

We further conducted two separate one-way ANOVAs with different feedback valences to test how positive and negative feedback may differently elicit neural activities in *a priori* ROIs under three contingencies. As shown in the Figure 3, positive feedback elicited significantly stronger ventral striatum activation (*p*_FDR-corr_ < 0.05, small-volume corrected) under the “gain” contingency than under the “lose” (*t*_(15)_ = 2.23, *p* = 0.04) and the “combined” (*t*_(15)_ = 5.15, *p* < 0.01) contingencies. Meanwhile, when participants received negative feedback, the ventral striatum (*p*_FDR-corr_ < 0.05, small-volume corrected) was significantly less deactivated under the “gain” (*t*_(15)_ = 5.50, *p* < 0.01) and “lose” (*t*_(15)_ = 3.94, *p* < 0.01) contingencies than under the “combined” contingency (see Figure 3). In addition, the dorsal amygdala showed significantly higher activation (*p*_FDR-corr_ < 0.05, small-volume corrected) under the “gain” (*t*_(15)_ = 4.23, *p* < 0.01) and the “lose” (*t*_(15)_ = 4.16, *p* < 0.01) contingencies than under the “combined” contingency when participants received negative feedback (see Figure 3). Table 2 presents detailed information about the activated brain regions from one-way ANOVA analyses.

**Table 2:** **Activated brain regions from one-way ANOVA analyses with different feedback valence**.

Brain regions	BA	R/L	Cluster	MNI Coordinates			*z*-value
				*x*	*y*	*z*	
**Positive feedback**							
Ventral striatum		R	13	14	12	−18	3.23
**Negative feedback**							
Ventral striatum		L	24	−16	8	−4	3.46
Amygdala		L	24	−30	−6	−8	3.09

*Note. Only regions of interest survived from small-volume correction at p_FDR-corr_ < 0.05 are presented. BA: Brodmann’s area; R/L: right or left hemisphere*.

## Discussion

We examined the effects of different types of reward contingencies on emotional and motivational responses during feedback processing by comparing the brain activation in several reward-sensitive regions. We found differential pattern of neural activities in the ventral striatum and the amygdala depending upon the type of reward contingency.

First, significant difference in the ventral striatum activation was observed across the three contingencies during overall feedback processing. Functional ROI results indicate that the ventral striatum showed positive activation only under the “gain” contingency and was deactivated under the “lose” and the “combined” contingencies. Moreover, two separate one-way ANOVAs with different feedback valences revealed that when participants received positive feedback, the ventral striatum showed significantly stronger activation under the “gain” contingency than under the other two contingencies. On the other hand, the ventral striatum was less deactivated under the “gain” contingency than under the “combined” contingency in response to negative feedback. The ventral striatum is known as the main reward area responsible for hedonic experience and its activation in response to a variety of reward has been reported (e.g., Delgado et al., 2000; Knutson et al., 2001a; O’Doherty et al., 2002; Delgado, 2007). At the same time, it is also known that reward omission or punishment results in deactivation in the ventral striatum (Knutson et al., 2001b; Pagnoni et al., 2002; O’Doherty et al., 2003). Findings from the present study suggest that compare to the “lose” and the “combined” contingencies, the “gain” contingency might be more likely to help individuals generate positive affect when receiving positive feedback and resist negative affect when receiving negative feedback. Based on a neuroscientific model of motivational process, Kim (2013) highlighted the important role of the ventral striatum during the motivation generation phase because it plays an essential role in generating approach behavior. We observed that feedback, especially the positive feedback, recruited the ventral striatum under the “gain” contingency. Because the “gain” contingency typically creates promotion focus which leads to eagerness (Higgins, 2000), positive feedback in the “gain” contingency could be a powerful incentive to enhance motivation.

Second, differential activation patterns of the amygdala were witnessed across the three reward contingencies. Overall feedback produced significantly higher activation in the bilateral amygdala under the “gain” and the “lose” contingencies than under the “combined” contingency. It is well documented that the amygdala plays an important role in processing negative and unpleasant emotions, such as fear and disgust (see Calder et al., 2001; Davis and Whalen, 2001 for review). Yet recent large meta-analyses of PET and fMRI studies on emotional processing have shown that the amygdala responded not only to negatively evaluated stimuli but also to positively evaluated stimuli (e.g., Sergerie et al., 2008; Mende-Siedlecki et al., 2013). Furthermore, accumulated evidence has pointed out that the amygdala plays a more critical role in coding stimulus salience than its valence (e.g., Costafreda et al., 2008; Mende-Siedlecki et al., 2013). Findings from the present study are consistent with this perspective, since the emotional facial feedback elicited significant activation of the bilateral amygdala. In addition, our results suggest that the identical feedback might be perceived as having different salience under different contingencies. The same feedback was perceived more salient in the promotion-focused “gain” contingency and the prevention-focused “lose” contingency than in the “combined” contingency. In particular, negative feedback selectively elicited stronger activation in the dorsal amygdala under the “gain” and “lose” contingencies. Whalen et al. (2001, 2013) have pointed out that the dorsal part of the amygdala is the key brain structure in encoding and evaluating negative stimuli, and subsequently sending vigilant signals to the cortical structures. Thus, the higher dorsal amygdala activation during negative feedback processing suggests that participants may become more vigilant under the “gain” and “lose” contingencies when having received negative feedback.

In addition, we also found that the sgACC showed different activation patterns across the three contingencies during overall feedback processing. It positively activated only under the “lose” contingency but deactivated under the “gain” and the “lose” contingencies. Interestingly, although the sgACC activation was witnessed from the main effect of contingency (regardless of the feedback valence), we found significant activation in the corresponding sgACC region (MNI coordinates: *x*, *y*, *z* = −10, 22, −10, 17 voxels, *p* < 0.005, uncorrected) only in the one-way ANOVA with negative feedback. Moreover, the activation pattern of the sgACC in the one-way ANOVA with negative feedback was exactly the same as the pattern observed in the factorial ANOVA. The sgACC is a well-documented brain region that engages in negative mood, particularly sadness (see Phan et al., 2002 for a review). Quantitative meta-analysis about neural correlates of basic emotions also revealed that sadness consistently activated the sgACC (Vytal and Hamann, 2010). Furthermore, personality-dependent activation in the sgACC has been found to be strongly linked to trait levels of anxiety (Haas et al., 2007). Significantly higher activations in the amygdala and the sgACC under the “lose” contingency suggest that participants might be more likely to experience negative emotion (such as sadness and anxiety) under the “lose” contingency, especially when having received negative feedback.

There was also a significant main effect of feedback on the lateral OFC in the 2 × 3 ANOVA analysis. Specifically, positive feedback has produced significantly stronger activation in the lateral OFC than negative feedback. The OFC is the critical brain region for value judgment (Grabenhorst and Rolls, 2011) and is sensitive in comparing relative value and responds only to preferred stimuli (Tremblay and Schultz, 1999). Furthermore, some researchers have suggested a medial-lateral distinction within the OFC, in which reward selectively recruits the medial OFC activation whereas punishment selectively recruits the lateral OFC activation (e.g., O’Doherty, 2007). However, in a recent meta-analysis, Sescousse et al. (2013) reported that both primary and secondary rewards consistently elicited activation in the lateral OFC. Similar findings have also been found in several empirical studies which both the medial and lateral OFC equally responded to both reward and punishment (e.g., Breiter et al., 2001; Elliott et al., 2003). These inconsistent results suggest that the medial-lateral dissociation between reward and punishment within the OFC needs further investigation.

Taken together, the findings of this study have practical implications for designing reward contexts that could beget positive affect and enhance individuals’ motivation. Depending upon the reward contingency, individuals could perceive an identical feedback differently and in turn experience different emotions and motivations. Among the three types of contingencies, we recommend implementing the “gain” contingency, in which a reward is given for success and no punishment is given for failure, because it shows the most adaptive pattern of emotional and motivational responses to both positive and negative feedback. Our interview data support this argument as most of the participants felt more satisfied with the “gain” contingency. Among the sixteen participants, nine rated the “gain” contingency, four rated the “combined” contingency, and only three rated the “lose” contingency as most satisfactory.

Several limitations of the present study as well as suggestions for future research need to be addressed. First, we used bogus feedback regardless of participants’ actual performance. Although all the participants believed that the feedback was based on their actual performance, it would be ideal for future research to use real feedback based on participants’ actual performance. Second, it would be worthwhile to further investigate if verbal feedback without a monetary reward would have a similar effect because verbal praise and punishment are more frequently used in educational settings.

## Conclusion

The present study investigated individuals’ emotional and motivational responses to three different types of reward contingencies during feedback processing. It contributes to the existing literature by demonstrating that contextual effect of reward could elicit distinct neural activities during feedback processing. In particular, the results indicate that the “gain” contingency is more likely to produce positive affect and maintain individuals’ motivation. Therefore, we suggest implementing reward/punishment systems based on the “gain” contingency to maintain individuals’ motivation during task performances.

## Conflict of interest statement

The authors declare that the research was conducted in the absence of any commercial or financial relationships that could be construed as a potential conflict of interest.
